# Large-Scale Clinical Evaluation of Rapid Blood Culture Identification Panels for Bloodstream Infections at a Tertiary Hospital

**DOI:** 10.3390/diagnostics13061177

**Published:** 2023-03-19

**Authors:** Min-Kyung So, Soo-Kyung Kim, Hae-Sun Chung, Ji-Yun Bae, Miae Lee

**Affiliations:** 1 Department of Laboratory Medicine, Ewha Womans University College of Medicine, Seoul 07985, Republic of Korea; 2 Department of Internal Medicine, Ewha Womans University College of Medicine, Seoul 07985, Republic of Korea

**Keywords:** bloodstream infections, FilmArray, blood culture identification

## Abstract

The prompt implementation of optimal antibacterial therapy through the rapid identification of the causative organisms is essential for improving outcomes for critically ill patients with bloodstream infections. We evaluated the clinical performance of the FilmArray blood culture identification (BCID) panel for rapidly identifying causative pathogens in the bloodstream using large-scale clinical samples. We analyzed the results of identification using a BCID panel performed on 2005 positive blood culture bottles from September 2019 to June 2022. Pathogen detection efficiency and interval from Gram staining to identification using the BCID panel were compared to those of conventional identification systems—VITEK MS MALDI-TOF Mass Spectrometer and Vitek2—and antibiotic susceptibility testing—Vitek2. We detected 2167 isolates from 2005 positive blood culture bottles. In these isolates, the BCID panel showed 93% full agreement—both organisms and antimicrobial resistance genes were matched, and no off-target organisms were detected. Species-level discordance was found in 0.6% of tests. Sixty-five isolates (3.0%) were only detected by BCID, whereas 22 isolates (1.0%) from the on-target panel were not detected by BCID. This large-scale study demonstrated that the BCID panel was a reliable and rapid identification method for directly identifying bloodstream pathogens in a positive blood culture.

## 1. Introduction

Bloodstream infection (BSI) is a critical cause of high mortality, morbidity, and increasing the duration of hospitalization and associated costs [1,2,3]. According to the Korea Sepsis Alliance’s 6-month research, the overall mortality rate owing to sepsis was 28.1%, with the hospital mortality rate of hospital-occurring sepsis being particularly high at 39.0% [4]. The mortality caused due to BSIs can be reduced through the prompt administration of targeted antibiotic therapy [3]. Clinical microbiology laboratories should be consistent with developing strategies to optimize the use of antimicrobial agents that have the potential to improve patient outcomes and reduce hospital costs [5].

Multiplex polymerase chain reaction (mPCR) panels, also known as syndrome panels, are considered a principal technology in the many strategies that integrate tests for multiple pathogens and/or antimicrobial resistance genes into a single test, providing rapid results for multiple targets [6,7]. The introduction of the mPCR panel in clinical microbiology laboratories is expected to improve patient care and clinical workflow, as well as reduce hospitalization costs by reducing diagnosis and decision-making time [8,9,10,11]. 

Several federal drug administration-approved mPCR panels have been commercialized as a technology that can quickly identify the causative bacteria and detect resistance genes for BSIs [12]. These panels are designed to simultaneously target bacteria, fungi, and antibiotic-resistance genes. Usually, the processing time is 0.5–8 h, which is faster than the time required for identification by conventional culture-based methods [12]. These panels can also provide critical antimicrobial susceptibility information using the technique for detecting antimicrobial resistance genes. 

For the rapid identification of etiologic BSI organisms, we introduced the FilmArray Blood Culture Identification (BCID) panel (BioFire Diagnostics, Salt Lake City, UT, USA)—an mPCR panel that automatically reads the results directly from positive blood cultures—in our institute. The panel is designed to simultaneously identify 24 microorganisms (eight gram-positive groups, 11 gram-negative groups, and five yeast groups) and three antimicrobial-resistance genes (*mecA*, *vanA/B*, and *bla*KPC) [13]. Before this panel can be used as an auxiliary tool for accurate clinical decisions and treatment policies, it is necessary to check its performance in identifying pathogens using various clinical samples and recognize its limitations. 

We herein evaluated the clinical performance of the BCID panel using 2005 positive blood culture samples compared to that of conventional blood culture and an antibiotic susceptibility test system. This report summarizes the results obtained three years after the BCID panel’s implementation. To our knowledge, the BCID panel results comprise the highest number of clinical samples analyzed from one institute.

## 2. Materials and Methods

### 2.1. Process of Blood Culture and BCID Panel Testing

This study retrospectively analyzed the data of patients from all age ranges from both conventional blood culture and BCID panel testing at a tertiary hospital comprising 680 beds in South Korea from September 2019 to June 2022. This study was approved by the Research Ethics Committee of the Ewha Womans University (approval number: EUMC2022-07-034). All microbiological analyses were performed in the Laboratory Medicine Department of the hospital. The flow of blood culture tests performed by the Department of Laboratory Medicine in the hospital to evaluate BCID performance was as follows: When a positive signal was detected in the collected blood culture bottle, Gram staining was performed, and the result was brought to the attention of the physician by phone within 1–2 working hours. Simultaneously, the results were added to the electronic medical records (EMR). After confirming the Gram staining results, an order for BCID was placed via a text message to the physician. Blood cultures identified during the night, Saturday afternoon, or Sunday afternoon were maintained in the incubator until working hours. Afterward, the BCID panel test was performed along with the conventional blood culture methods only for samples for which an order for BCID was obtained. When several positive culture samples were obtained from the same patient, BCID testing was performed on the sample in the bottle that revealed the first positive signal. In the laboratory, BCID testing was conducted during working hours (8 a.m.–5 p.m.) from Monday to Saturday noon. Outside of working hours, most blood culture specimens did not receive the BCID order, so the BCID panel test could not be performed. Through the aforementioned protocol, 2005 positive blood culture samples were assigned a BCID order, and all cultures for this test were compared to those evaluated using the culture-based identification method.

### 2.2. Conventional Identification and Antimicrobial Susceptibility Methods

For adults, aerobic and anaerobic blood culture bottles were collected. For children, blood culture tests were performed using only aerobic culture bottles. Blood samples were cultured using a BACT/ALERT VIRTUO automated blood culture system (bioMérieux, Marcy l’Etoile, France) according to the manufacturer’s instructions. The samples were cultured until a positive signal was obtained or for a period of five days. When the blood culture bottle produced a positive signal, it was removed from the system, and the organisms were evaluated using Gram staining.

Blood culture-positive samples grown in aerobic bottles were subcultured onto 5% sheep blood and MacConkey agars. If the anaerobic culture bottle indicated a positive signal, a subculture was additionally performed on Brucella agar. The subcultured bacteria were used for microbial identification and antimicrobial susceptibility tests (AST). Microorganisms grown on agar plates were identified mostly through a VITEK MS MALDI-TOF Mass Spectrometer (MALDI-TOF, bioMérieux, Marcy l’Etoile, France). The VITEK2 identification system (bioMérieux, Marcy l’Etoile, France) was also used in some cases. Results were confirmed by catalase, oxidase, indole, the Christie–Atkins–Munch–Peterson (CAMP) test, and the optochin test according to the Clinical and Laboratory Standards Institute (CLSI) guideline. The results of conventional AST were confirmed using the VITEK2 Susceptibility system (bioMérieux, Marcy l’Etoile, France), and assessments were performed using the susceptible, intermediate, or resistant categories according to the CLSI guideline. Vancomycin-resistant Enterococci (VRE) were also confirmed through the disk diffusion method. Resistance for imipenem in *Proteus* isolates was confirmed using a disk diffusion method. When carbapenem-resistant Enterobacteriaceae were detected, a modified Hodge test and carbapenemase inhibition test were performed. 

### 2.3. BCID Panel Testing Method

BCID analysis was performed using multiplex PCR according to the manufacturer’s instructions. Briefly, upon confirmation of a positive signal in the blood culture bottle, 100 μL of the positive culture medium was diluted in 500 μL of sample buffer, and 300 μL of this sample solution was subsequently injected into the BCID pouch. Thereafter, extraction, amplification, detection, and analysis were performed in a fully automated method on a BioFire FilmArray instrument (BioFire Diagnostics, Salt Lake City, UT, USA). Each pouch contains two internal running controls; if either control fails, the result is displayed as “invalid”. If a clinician placed the order, BCID panel testing was performed on specimens with Gram staining results, including gram-positive bacilli, even in cases where the panel lacked the bacterial target. 

### 2.4. Evaluation of the Agreement between Conventional Methods and BCID

The accuracy of the BCID panel was compared with that of conventional blood culture methods. “Full agreement” was considered to have been reached when the results of the antibiotic susceptibility and those of the antibiotic resistance genes matched with the target bacteria or when bacteria not included in the BCID panel targets were not detected. Discrepancies at the species level and discordance between antimicrobial susceptibility results and antibiotic resistance genes were deemed to indicate a “Mismatch”. “Detection failure” meant that the target organism identified by blood culture was not identified by BCID, even though the organism was included among the targets. Organisms not detected in blood culture but detected using the BCID panel were classified as “Over-detection”. If the BCID manufacturer’s insert mentions bacteria as undetectable organisms, even those in the same genus were considered off-target.

### 2.5. Additional Investigations for Antibiotic Susceptibility Discrepancies and blaKPC Gene Confirmation

Additional investigations were performed in case of discrepancies between the antibiotic susceptibility results and genes for *vanA/B* and *bla*KPC. If the vancomycin antibiotic susceptibility results did not match the vanA/B genes from BCID, we investigated whether VRE was detected through the surveillance test in the rectal swab of each patient within two weeks from the BCID-performance date. Moreover, in cases where the blaKPC did not match with a carbapenem (ertapenem and/or imipenem) AST as well as blaKPC detected samples, we performed the Xpert Carba-R assay (Cepheid, Sunnyvale, CA, USA) on the same sample. Furthermore, even if blaKPC was consistently detected with AST, we confirmed its presence using Xpert Carba-R. The Xpert Carba-R assay tests for the presence of five common carbapenemase genes—*bla*KPC, *bla*NDM, *bla*VIM, *bla*IMP-1, and *bla*OXA-48—according to the manufacturer’s instructions. We did not conduct any further investigation on the *mecA*-related discrepancies.

### 2.6. Comparison of Reporting Time of Each Method in the Blood Culture System

For each sample analyzed, the following times were checked: time taken from receipt of blood culture to the reporting of Gram staining results of the blood culture bottles with a positive signal (h); time taken from the reporting of Gram staining results to (1) receipt after the BCID test order time (h), (2) to BCID test-performing time taken to report the results (h), (3) to time reporting interim identification results using conventional methods (MALD-TOF), and (4) to time to report the final identification and antibiotic susceptibility results. The time at which each result was reported was based on that inserted in the EMR.

## 3. Results

### 3.1. Identified Organisms

During the study period, 113,471 blood bottles, at an average of 3337 bottles/month, were collected and incubated, of which 9565 samples exhibited growth signals. We performed BCID panel testing on 2005 (21%) of these samples simultaneously. We detected 2167 isolates (BCID, 1965; conventional methods, 2102) from 2005 positive blood culture bottles (Table 1). Except for *Haemophilus influenzae* and *Neisseria meningitidis*, all targets of the BCID panel were detected from patients’ blood cultures. Of the isolates, 1965 (90.7%) microorganisms were detected by the BCID panel, and 202 (9.3%) microorganisms were detected only by conventional methods. The organisms most frequently found in this study comprised coagulase-negative Staphylococci (CoNS), followed by *Escherichia coli*.

### 3.2. Multi-Microorganism Detectability

Of the 2005 BCID results obtained, mono-microbial isolates were detected in 1690 (84.3%) bottles, multi-microbial isolates in 132 (6.6%) bottles, and no isolates in 183 (9.1%) bottles (Table 2). As per the BCID testing results, 132 (6.6%) bottles had multi-microbial growth, whereas 87 (4.3%) had multi-microbial growth as detected by conventional methods. Up to four organisms were detected in one bottle. Among the 141 multi-microbial growth bottles, 71 (50.4%) bottles showed the same number of microorganisms in both BCID and conventional methods, 58 (41%) bottles were detected to have more microorganisms in BCID, and only 12 (8.5%) had higher numbers of organisms detected through conventional methods. Interestingly, the most additionally detected organism by BCID was *Proteus* (31 cases), which was only detected by BCID in multi-microbial growth bottles tested from May 2020 to October 2020. 

### 3.3. Agreement between Conventional Methods and BCID

Table 3 shows the comparison between conventional methods and BCID in detail. Based on the 2167 isolates, the BCID panel showed full agreement in 2016 isolates (93.0%), matching both organisms and antimicrobial resistance genes or where off-target organisms were not detected. Species-level discordance was found in 13 isolates (0.6%). Sixty-five isolates (3.0%) were only detected by BCID, whereas 22 isolates (1.0%) from the on-target panel were not detected by BCID. Of the detected organisms, 52 isolates (2.4%) showed discrepancies between the antimicrobial resistance genes detected by BCID and antimicrobial susceptibility results from the Vitek2 system. When subdivided into groups, the full agreement rates obtained through BCID were 93.7% and 92.5% for gram-positive and gram-negative bacteria, respectively, whereas yeast showed a low agreement of 85.7%. In the yeast group, except for one case of *Candida parapsilosis*, all yeasts isolated by conventional methods were identically detected on a species level in the BCID panel. However, ten yeast isolates (13.0%) were additionally identified along with other organisms in BCID only. Of these additional isolates, four were identified along with yeast, and six were identified along with the bacteria *B. cereus*, *C. striatum*, *S. epidermidis*, *E. coli*, and *P. mirabilis*.

### 3.4. Off-Panel Organisms

A total of 180 microorganisms that were not included in the BCID panel were isolated. Of these, 122 (67.8%) were gram-positive, 55 (30.5%) were gram-negative, and three (1.7%) were yeasts (Table 4). The most common off-target organisms not detected by BCID were—in order—*Corynebacterium* spp., *Bacillus* spp., *Staphylococcus pettenkoferi*, *Bacteroides* spp., and *Stenotrophomonas maltophilia*, *Micrococcus luteus*, and *Clostridium* spp. Table 4 summarizes the list of isolates detected by conventional methods in this study that are not included in the BCID panel.

### 3.5. Evaluation of Antimicrobial Resistance Genes Detected by BCID Compared to Those Detected through the Conventional Sensitivity Test

In our study, the antimicrobial resistance genes, *bla*KPC, *vanA/B*, and *mecA*, included in the BCID panel were detected as follows: of 2167 isolates, 84 (3.9%) had *vanA/B*, 725 (33.5%) had *mecA*, and 11 (0.5%) had *bla*KPC. The *vanA/B* gene was observed in 82 *E. faecium* and two *E. faecalis* cultures. The two isolates (one *S. aureus* and one CoNS) in which *MecA* was detected could not be identified in culture and were excluded from Table 5. Among the 723 isolates with mecA, 63 isolates (8.7%) comprised *S. aureus*, whereas the others comprised CoNS (660 isolates). All samples in which *bla*KPC was detected were of *K. pneumoniae* (Table 5). 

The concordance between the samples detected with *vanA/B* in the BCID panel and conventional AST was 97.2%. Samples in which BCID did not detect *vanA/B* were all shown to be sensitive to vancomycin. However, *vanA/B* was detected in five cases of *Enterococcus* (*E. faecalis*, n = 1; *E. faecium*, n = 4) that were susceptible to vancomycin, resulting in a discrepancy between the gene and the phenotype. Each sample was detected in a different patient. Interestingly, the three patients with a discrepancy in results had their VRE detected through a rectal swab for a surveillance test within a short period.

For *mecA*, 43 isolates (4.7% of CoNS and *S. aureus*)—38 CoNS samples and five *S. aureus* samples—showed discrepancies. Our data revealed that *mecA* was not detected; however, there were 13 oxacillin-resistant organisms in which *mecA* was detected and 30 oxacillin-sensitive isolates. The mismatch rate between *mecA* and oxacillin in the CoNS samples was 4.9% (38/778), and the incompatibility rate for *S. aureus* samples was 3.8% (5/133). There was no significant difference between CoNS and *S. aureus* (*p*-value = 0.5813). We were unable to further study the *mecA* gene in cases of discrepancy for confirmation. 

All *blaKPC*-detected *K. pneumoniae* (11 isolates) showed resistance to carbapenems (both ertapenem and imipenem). However, five *Enterobacteriaceae* for which *bla*KPC was not identified showed resistance to ertapenem and/or imipenem. In the additional molecular testing by the Xpert Carba-R test, which can simultaneously detect the carbapenemase genes—*bla*KPC, *bla*NDM, *bla*VIM, *bla*IMP-1, and *bla*OXA-48–the *bla*NDM gene was detected from one isolate (*E. cloacae* complex), and two isolates—*K. pneumoniae* and *E. coli*—were not detected to have any of the genes. The other two isolates were *Proteus*, which showed imipenem resistance and ertapenem sensitivity.

### 3.6. Turnaround Time

**Figure 1:**
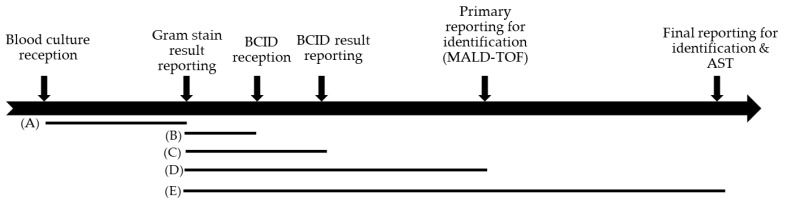
Schematic diagram of the blood culture reporting process of conventional and BCID blood cultures in our institution.

Starting from the Gram staining reporting time, the BCID reporting time took a median of 5.7 h (Table 6, Figure 1). Of this, it took 4.1 h to contact the clinic and obtain a BCID panel order. More specifically, it took 1.6 h from order reception to the reporting of BCID panel results. The time was 21 h faster than the interim reporting time (27.0 h) using MALDI-TOF. A median of 48.5 h was taken for the final reporting of results, with identification and antimicrobial susceptibility, and the difference in turnaround time between the BCID panel and final culture results was approximately 42.8 h. 

## 4. Discussion

In this paper, we evaluated the BCID panel, a multi-target PCR technique, and compared its results with those of conventional comparator methods, including a MALDI-TOF identification and a Vitek2 antimicrobial susceptibility assay. In our data, among 2167 organisms identified by conventional methods or BCID, 1965 (90.7%) were detectable by BCID. Compared with conventional culture methods, BCID panels showed 93% full agreement—both organisms and antibiotics genes were matched, or no off-target organisms were detected. Species-level discordance was found in 0.6% of tests. The full agreement rate in yeast groups was lower (85.7%) than that in other groups (93.7% in gram-positive and 92.5% in gram-negative samples). These results are consistent with those in earlier studies on yeast in clinical samples [14]. The additional isolates could be nonviable organisms or false detection. Resolving this question requires further investigation.

The identification of bacteria showed consistency but also discrepancies between the BCID antimicrobial resistance genes and antimicrobial susceptibility results of the conventional AST test in 2.4% of cases. Moreover, 3.0% of organisms were only detected by BCID, whereas BCID did not detect 1.0% of organisms from the on-target panel.

Of the 202 organisms from the 2167 organisms not detected by the BCID panel, 180 isolates (90%) were off-target organisms according to the manufacturer’s instructions. The most common off-target organisms not detected by BCID were—in order—*Corynebacterium*, *Bacillus*, *Staphylococcus pettenkoferi*, *Bacteroides*, *Stenotrophomonas maltophilia*, *Micrococcus luteus*, and *Clostridium*. Of these organisms, *Corynebacterium*, *Bacillus*, *Staphylococcus pettenkoferi*, and *Micrococcus luteus* were considered contaminant organisms that are usually not related to bacteremia [15,16]; therefore, their false-negative detection rate could be of little clinical significance. However, gram-negative bacteria, including *Stenotrophomonas maltophilia* and *Bacteroides*, and gram-positive bacteria, including *Clostridium*, could be clinically significant pathogens to patients [17]. Fortunately, the recent upgrade of the BCID2 panel format is expected to overcome the limitations of the current BCID panel by including *Bacteroides fragilis* and *Stenotrophomonas maltophilia* as targets among the gram-negative bacteria not included in the BCID panel [18,19]. However, *Clostridium*, which can still be clinically significant, was not added to the BCID2 panel; accordingly, additional consideration is needed.

Antimicrobial-resistance genes in the BCID panel include *bla*KPC, *mecA*, and *vanA/B* as targets. The detection of these genes is essential for the identification of antibiotic resistance genes. BCID results help provide important guidelines for promptly administering targeted antibiotic therapy in critically ill patients with sepsis. Molecular screening of antibiotic-resistance genes showed good concordance compared with the results from the BCID panel and VITEK 2 susceptibility test. Discordant results were present only for 2.8% of samples with *vanA/B*, 4.7% (4.9% of CoNS, 3.8% of *S. aureus*) of samples with *mecA*, and 0.9% of samples with *bla*KPC. This discrepancy could be because the BCID test uses a blood sample directly from the positive blood bottle. In contrast, the conventional AST methods used a single colony after subculture on an agar medium from a positive blood bottle. In our data, we observed five *Enterococcus* samples with *vanA/B* detection by BCID, which were sensitive to vancomycin in an AST test. Interestingly, three among five patients having a discrepancy in results were found to have VRE by surveillance tests through rectal swabs in the proximity period. Evidence suggests a mixed presence of VRE in blood culture bottle samples. A second possibility is that the antimicrobial-resistance gene included in the BCID panel may not be the causative gene for the phenotype. In our data, *bla*KPC was not identified in 5 of 500 samples of *Enterobacteriaceae* but was resistant to ertapenem or imipenem. When this sample was confirmed with the Xpert Carba-R assay, which can simultaneously detect the carbapenemase genes *bla*KPC, *bla*NDM, *bla*VIM, *bla*IMP, and *bla*OXA-48, one sample was detected to have *NDM*, and in two samples (*E. coli* and *K. pneumoniae*), the carbapenemase genes were not detected. The remaining two discordant specimens were *P. mirabilis*, which is known to show resistance to imipenem by mechanisms other than that for carbapenemase. With these five discordant samples analyzed, we were able to conclude that the *bla*KPC detection agreement is almost 100%. Moreover, a recent upgrade in the form of the BCID2 panel has been expanded to include additional carbapenemase genes, such as *bla*NDM, *bla*OXA-48-like, *bla*IMP, and *bla*VIM, which is expected to show better performance by broadening the scope of the limited antimicrobial resistance gene targets [18].

The BCID panel can detect multi-organism growth in culture bottles; however, there have also been false-positive results. Moreover, 6.5% (132 bottles) of positive cultures showed BCID-detectable multi-organisms containing up to four strains. Of these, 58 sample results from the BCID panel detected more organisms not detected by conventional methods. Of these, 31 samples with *Proteus* strains were detected with one or two other organisms. This result was obtained during a specific period from May 2020 to October 2020. From the bottles, we were unable to find clues regarding the presence of *Proteus* from the traditional culture method. Except for this period, there was no simultaneous detection of additional *Proteus* with other microorganisms. A study reported no false-positive results in 1568 clinical samples [14]; however, the manufacturer had reports of false detection of *Proteus* by BCID panels due to nonviable *Proteus* DNA contamination in certain blood culture media lots prepared by Bactec and BacT/Alert [20]. Accordingly, our false-positive results were also related to this issue.

Several previous studies using the BCID panel have been published. One study contained 2207 samples (1568 clinical samples and 639 seeded positive blood cultures) from eight centers [14]. Other than that, most of the previous studies included between 54 and 206 samples [13,21,22,23,24]. We compared our study to the Salimnia et al. study, which utilized a large number of samples to evaluate microorganism identification agreement rates [14]. Salimnia et al. found that the identification agreement rate for gram-positive bacteria was 95.6% (1494/1563), which is similar to our rate of 95.4% (excluding consideration of resistant genes). In the case of gram-negative bacteria, our identification rate was 93.1%, which was slightly lower than the 95.6% (1135/1187) reported by Salimnia et al. This discrepancy was attributed to the detection of a false positive, which was identified as Proteus in multi-organism detected bottles in our study. Furthermore, we observed that the identification rate for yeast was lower in our study compared to Salimnia et al.’s (85.7% vs. 96.1%, respectively). However, it is worth noting that seed samples accounted for the majority of yeast in their study, while only clinical samples were considered, resulting in a concordance rate of 85% (42/49), which is similar to our study. It should be noted that both studies showed discrepancies in additionally detected yeast cases. Studies that used BCID panels with small sample sizes reported detection rates ranging from 80.4% to 91.6% for all organisms in clinical performance (94.6–99.3% for the on-target organisms in the BCID panel) [13,21,22,23,24]. Compared with these papers, the number of clinical samples analyzed in 2005 positive blood culture bottles in our research was sufficiently large. Moreover, our data gave additional information on various on- and off-target isolates in each group compared to other studies. 

The cost and staffing requirements of the BCID system may vary depending on factors such as laboratory size and sample volume relative to MALDI-TOF or Vitek2. Although the BCID panel is more expensive than the unit price of reagents per sample for MALDI-TOF or Vitek2, total expenses may be reduced in the long run due to the prompt detection of bacteria by BCID, optimizing antibiotic use, and reducing hospital stays. Additionally, the simplicity of the BCID system and equipment may make it easier and less expensive to establish than MALDI-TOF or Vitek2, which require significant investment to build the initial system. Regarding staffing, the BCID system requires fewer personnel to operate due to its automation and minimal need for manual labor compared to traditional culture-based systems. Our laboratory did not require additional staffing to execute the BCID system.

This study has some limitations. Although, to our knowledge, our research was considered one of the largest clinical studies on the BCID panel, it was limited in identifying specific target organisms. Of the samples included, our samples did not include *Haemophilus influenzae* and *Neisseria meningitidis*, which are BCID target organisms, and there was only one *Streptococcus pyogenes* sample. Although these pathogens are clinically extremely rare as a cause of sepsis, the presence of pathogenic organisms is clinically significant. Therefore, further evaluations are warranted for rare target organisms. Second, several previous studies have shown that improving turnaround time through BCID has a positive clinical effect on shortening the time to optimize antibacterial treatment for patients with BSIs, especially those requiring antibiotic susceptibility testing and subsequent molecular screening [9,25,26]. However, this study has yet to evaluate the impact of this rapid reporting of results on patient health and health service outcomes in clinical settings, which remains a subject of further research. 

## 5. Conclusions

Our large-scale clinical study has shown that the BCID panel is highly concordant with conventional methods for directly identifying gram-positive and gram-negative bacteria and yeast in positive blood culture bottles. Furthermore, the panel is a useful tool for predicting critical antibiotic susceptibility outcomes. Additionally, our study demonstrated that the time required for identifying organisms in blood culture bottles could be significantly reduced with the use of the BCID panel. Based on this large-scale investigation, the BCID panel provides a reliable and rapid method for directly identifying bloodstream pathogens in positive blood cultures.

## Figures and Tables

**Table 1:** Prevalence of microorganisms from positive blood culture bottles detected with the BCID panel.

Microorganisms Detected by the BCID Panel	No.	Group% (Sub% *)
Gram-positive bacteria	1177	54.3% (100%)
*Enterococcus*	184	(15.6%)
*Listeria monocytogenes*	3	(0.3%)
*Staphylococcus* (CoNS)	780	(66.3%)
*Staphylococcus aureus*	135	(11.5%)
*Streptococcus* (not *S. agalactiae*, *S. pneumoniae*, or *S. pyogenes*)	53	(4.5%)
*S. agalactiae*	18	(1.5%)
*S. pneumoniae*	3	(0.3%)
*S. pyogenes*	1	(0.1%)
Gram-negative bacteria	715	33.0% (100%)
*Acinetobacter baumannii*	78	(10.9%)
*Haemophilus influenzae*	0	(0.0%)
*Neisseria meningitidis*	0	(0.0%)
*Pseudomonas aeruginosa*	28	(3.9%)
*Enterobacteriaceae* (not *E. cloacae*, *E. coli*, *K. oxytoca*, *K. pneumoniae*, *Proteus*, or *S. marcescens*)	19	(2.7%)
*E. cloacae* complex	18	(2.5%)
*E. coli*	335	(46.9%)
*K. oxytoca*	14	(2.0%)
*K. pneumoniae*	149	(20.8%)
*Proteus*	69	(9.7%)
*Serratia marcescens*	5	(0.7%)
Yeast	73	3.4% (100%)
*Candida albicans*	33	(45.2%)
*Candida glabrata*	9	(12.3%)
*Candida parapsilosis*	11	(15.1%)
*Candida tropicalis*	20	(27.4%)
Microorganisms detected only by conventional methods	202	9.3%
Total	2167	100.0%

Abbreviations: *S. agalactiae*, *Streptococcus agalactiae*; *S. pneumoniae*, *Streptococcus agalactiae*; *S. pyogenes*, *Streptococcus pyogenes*; *E. cloacae*, *Escherichia cloacae*; *E. coli*, *Escherichia coli*; *K. oxytoca*, *Klebsiella oxytoca*; *K. pneumoniae*, *Klebsiella pneumoniae*; BCID, blood culture identification. * Indicated percentage of each microorganism’s occupancy within the groups.

**Table 2:** Number of microorganisms isolated from positive blood culture bottles.

Number of Microorganisms Detected		No. of Bottles	% of Total Bottles
In BCID	In Conventional Methods		
None	One	183	9.1%
One	One	1681	84.3%
	Two	9	
Two	One	53 *	6.1%
	Two	67	
	Three	3	
Three	One	1	0.4%
	Two	4 *	
	Three	3	
Four	Four	1	<0.1%

* *Proteus* was identified in 31 bottles only by the BCID panel, demonstrating false-positive detection. Abbreviations: BCID, blood culture identification.

**Table 3:** Comparison of the identification of microbial pathogens from positive microbial blood cultures between the BCID panel and conventional culture methods.

	Comparison with Organisms Identified by Conventional Methods				
No. of Identified Organisms	Full Agreement	Mismatched		Over-Detection	Detection Failure
		Identification Matched/Resistance Gene Mismatched	Species Level Identification Mismatched		
Total (2167)	2015 (93.0%)	52 (2.4%)	13 (0.6%)	65 (3.0%)	22 (1.0%)
Gram-positive bacteria (1314)	1231 (93.7%)	47 (3.6%)	6 (0.5%)	15 (1.1%)	15 (1.1%)
*Enterococcus* (184)	175	5	-	4	2
*Listeria monocytogenes* (3)	3	-	0	0	0
*Staphylococcus* (CoNS) (780)	739	38	1	2	11
*S. aureus* (135)	125	4	4 §	2	1
*Streptococcus* * (Not *S.agalactiae*, *S.pneumoniae*, or *S.pyogenes*) (53)	46	-	1	6	1
*S. agalactiae* (18)	17	-	0	1	0
*S. pneumoniae* (3)	3	-	0	0	0
*S. pyogenes* (1)	1	-	0	0	0
No pathogen (off-panel organism)	122				
Gram-negative bacteria (776)	718 (92.5%)	5 (0.6%) □	7 (0.9%)	40 (5.2%)	6 (0.8%)
*Acinetobacter baumannii* (78)	78	0	0	0	2
*Haemophilus influenzae* (0)	0	0	0	0	0
*Neisseria meningitidis* (0)	0	0	0	0	0
*Pseudomonas aeruginosa* (28)	28	0	0	0	0
*Enterobacteriaceae* (Not *E. cloacae*, *E. coli*, *K. oxytoca*, *K. pneumoniae*, *Proteus* or *S. marcescens*) (20)	18	-	1	0	1
*E. cloacae* complex (18)	15	1	2	0	0
*E. coli* (335)	331	1	0	3	3
*K. oxytoca* (14)	13	0	1	0	0
*K. pneumoniae* (149)	140	1	3	5	
*Proteus* (69)	35	2	-	32	0
*Serratia marcescens* (5)	5	0			
No pathogen (off-panel organism)	55				
Yeast (77)	66 (85.7%)	-	0 (0.0%)	10 (13.0%)	1 (1.3%)
*Candida albicans* (33)	30	-	0	3	0
*Candida glabrata* (9)	7	-	0	2	0
*Candida parapsilosis* (11)	6	-	0	5	1
*Candida tropicalis* (20)	20	-	0	0	0
No pathogen (off-panel organism)	3				

* In this group, *S. auricularis* and *S. pettenkoferi*, listed as undetectable organisms on the BCID manufacturer’s insert, were considered off-target organisms. § Among the four isolates, one (identified as *S. capitis*) was found to have discordant resistance genes and AST results. □ These data were presented in the results compared with conventional AST methods. The discordant results did not reflect the subsequent Xpert Carba-R assay. Investigations of the discordant results are described in Table 4. Abbreviations: *S. aureus*, *Staphylococcus aureus*; *S. agalactiae*, *Streptococcus agalactiae*; *S. pneumoniae*, *Streptococcus agalactiae*; *S. pyogenes*, *Streptococcus pyogenes*; *E. cloacae*, *Escherichia cloacae*; *E. coli*, *Escherichia coli*; *K. oxytoca*, *Klebsiella oxytoca*; *K. pneumoniae*, *Klebsiella pneumoniae*; BCID, blood culture identification.

**Table 4:** BCID off-panel organisms identified by conventional methods.

BCID	Isolates Identified by Conventional Methods (No.)		
Off-target	Gram-positive bacteria (122)	Gram-negative bacteria (55)	Yeast (3)
	*Actinomyces neuii* (1) *Actinotignum schaalii* (1) *Aerococcus urinae* (1) *Bacillus* spp. (23) *Bifidobacterium* sp. (1) *Brevibacterium luteolum* (1) *Clostridium* spp. (8) *Corynebacterium* spp. (28) *Dermabacter hominis* (2) *Eggerthia catenaformis* (1) *Eubacterium lentum* (1) *Kocuria* spp. (2) *Lactobacillus* spp. (4) *Lactococcus lactis* (3) *Leuconostoc* sp. (2) *Micrococcus luteus* (12) *Pediococcus pentosaceus* (1) *PeptoStreptococcus micros* (3) *Robinsoniella peoriensis* (1) *Rothia mucilaginosa* (1) *Staphylococcus auricularis* (2) ** Staphylococcus pettenkoferi (22) ** Unidentified G(+)rods (1)	*Acinetobacter* spp. (5) *Aeromonas* spp. (2) *Bacteroides* spp. (17) *Burkholderia cepacia* (4) *Cupriavidus pauculus* (1) *Fusobacterium* spp. (2) *Moraxella* sp. (4) ** Prevotella spp. (2) *Providencia* spp. (2) ** Pseudomonas putida (1) *Roseomonas* spp. (2) *Stenotrophomonas maltophilia* (11) Unidentified G(-) rods (1) *Veillonella* sp. (1)	*Candida nivariensis* (1) *Saccharomyces cerevisiae* (2)

** Mentioned as undetectable organisms on the BCID manufacturer’s insert; even microorganisms included in the genus level were considered off-target. BCID, blood culture identification.

**Table 5:** Investigation of antimicrobial resistance genes as detected by BCID in comparison to using AST.

BCID			Vitek 2	No. (%)	Concordance (%)	Additional Information
Isolates (No.)	Gene	Detection	AST Results			
*Enterococcus* (180)	*vanA/B*	Not detected	S (vancomycin)	96 (53.3%)	Matched (97.2%)	
		Detected	R (vancomycin)	79 (43.9%)		
		Detected	S (vancomycin)	5 (2.8%)	Mismatched (2.8%)	Growth of VRE (3)/ no growth of VRE (1)/Not tested (1) §
*Staphylococcus* (CNoS) (778)	*mecA*	Detected	R (oxacillin)	632 (81.2%)	Matched (95.1%)	
		Not detected	S (oxacillin)	108 (13.9%)		
		Detected	S (oxacillin)	28 (3.6%)	Mismatched (4.9%)	
		Not detected	R (oxacillin)	10 (1.3%)		
*S. aureus* (133)	*mecA*	Detected	R (oxacillin)	61 (45.9%)	Matched (96.2%)	
		Not detected	S (oxacillin)	67 (50.4%)		
		Detected	S (oxacillin)	2 (1.5%)	Mismatched (3.8%)	
		Not detected	R (oxacillin)	3 (2.3%)		
*Enterobacteriaceae* (550)	*bla*KPC	Not detected	S (ertapenem and imipenem)	534 (97.1%)	Matched (99.1%)	
		Detected	R (ertapenem and imipenem)	11 (2.0%)		*bla*KPC (11) □
		Not detected	R (ertapenem and/or imipenem)	5 (0.9%)	Mismatched * (0.9%)	*bla*NDM (1) □/Not detected (2) □, *Proteus* (2)

Abbreviations: AST, Antimicrobial susceptibility test; R, resistant; S, sensitive; VRE, vancomycin-resistant *Enterococcus*. § Surveillance test from rectal swab, □ Confirmed by Xpert Carba-R assay, * Final concordance according to the additional information: Matched.

**Table 6:** Turnaround time from positive blood culture bottles using conventional methods or BCID.

Time from	Time to	Median, Hours (95% CI)	*
Blood culture reception time	Gram staining result reporting time	24.18 (22.96–24.98)	(A)
Gram staining result reporting time	BCID reception time	4.06 (3.49–5.21)	(B)
	BCID result reporting time	5.71 (5.27–7.53)	(C)
	Blood culture primary reporting time (Identification by MALDI-TOF)	26.95 (26.67–27.05)	(D)
	Blood culture final reporting time (Identification & sensitivity by Vitek2)	48.45 (48.28–48.66)	(E)

* (A)–(E) are shown in the schematic diagram of Figure 1.

## Data Availability

Data for this study, though not available in a public repository, will be made available upon reasonable request.

## References

[1] Chertoff J., Ataya A. (2017). The Timing of Early Antibiotics and Hospital Mortality in Sepsis: Playing Devil’s Advocate. Am. J. Respir. Crit. Care Med..

[2] Van Heuverswyn J., Valik J.K., Desiree van der Werff S., Hedberg P., Giske C., Naucler P. (2023). Association Between Time to Appropriate Antimicrobial Treatment and 30-day Mortality in Patients with Bloodstream Infections: A Retrospective Cohort Study. Clin. Infect. Dis..

[3] Goto M., Al-Hasan M.N. (2013). Overall burden of bloodstream infection and nosocomial bloodstream infection in North America and Europe. Clin. Microbiol. Infect..

[4] Kim S.N., Bahk H.J., Lee H.M. (2020). The Result of an In-depth Investigation to Improve the Management of Sepsis in Korea (1st year). Public. Health Wkly. Rep..

[5] Perez K.K., Olsen R.J., Musick W.L., Cernoch P.L., Davis J.R., Peterson L.E., Musser J.M. (2014). Integrating rapid diagnostics and antimicrobial stewardship improves outcomes in patients with antibiotic-resistant Gram-negative bacteremia. J. Infect..

[6] Dien Bard J., McElvania E. (2020). Panels and Syndromic Testing in Clinical Microbiology. Clin. Lab. Med..

[7] Dumkow L.E., Worden L.J., Rao S.N. (2021). Syndromic diagnostic testing: A new way to approach patient care in the treatment of infectious diseases. J. Antimicrob. Chemother..

[8] Nasef R., El Lababidi R., Alatoom A., Krishnaprasad S., Bonilla F. (2020). The Impact of Integrating Rapid PCR-Based Blood Culture Identification Panel to an Established Antimicrobial Stewardship Program in the United Arab of Emirates. Int. J. Infect. Dis..

[9] Britt N.S., Khader K., He T., Willson T.M., Effiong A., Timbrook T.T., Potter E.M., Lodise T.P. (2023). Examining the clinical impact of rapid multiplex polymerase chain reaction-based diagnostic testing for bloodstream infections in a national cohort of the Veterans Health Administration. Pharmacotherapy.

[10] Bookstaver P.B., Nimmich E.B., Smith T.J., Justo J.A., Kohn J., Hammer K.L., Troficanto C., Albrecht H.A., Al-Hasan M.N. (2017). Cumulative Effect of an Antimicrobial Stewardship and Rapid Diagnostic Testing Bundle on Early Streamlining of Antimicrobial Therapy in Gram-Negative Bloodstream Infections. Antimicrob. Agents Chemother..

[11] Timbrook T.T., Morton J.B., McConeghy K.W., Caffrey A.R., Mylonakis E., LaPlante K.L. (2017). The Effect of Molecular Rapid Diagnostic Testing on Clinical Outcomes in Bloodstream Infections: A Systematic Review and Meta-analysis. Clin. Infect. Dis..

[12] Peker N., Couto N., Sinha B., Rossen J.W. (2018). Diagnosis of bloodstream infections from positive blood cultures and directly from blood samples: Recent developments in molecular approaches. Clin. Microbiol. Infect..

[13] Southern T.R., VanSchooneveld T.C., Bannister D.L., Brown T.L., Crismon A.S., Buss S.N., Iwen P.C., Fey P.D. (2015). Implementation and performance of the BioFire FilmArray(R) Blood Culture Identification panel with antimicrobial treatment recommendations for bloodstream infections at a midwestern academic tertiary hospital. Diagn. Microbiol. Infect. Dis..

[14] Salimnia H., Fairfax M.R., Lephart P.R., Schreckenberger P., DesJarlais S.M., Johnson J.K., Robinson G., Carroll K.C., Greer A., Morgan M. (2016). Evaluation of the FilmArray Blood Culture Identification Panel: Results of a Multicenter Controlled Trial. J. Clin. Microbiol..

[15] Hall K.K., Lyman J.A. (2006). Updated review of blood culture contamination. Clin. Microbiol. Rev..

[16] Ugaban K.B., She R.C. (2022). The clinical significance of staphylococcus pettenkoferi: A retrospective review at a tertiary care medical center. Diagn. Microbiol. Infect. Dis..

[17] Rechner P.M., Agger W.A., Mruz K., Cogbill T.H. (2001). Clinical features of clostridial bacteremia: A review from a rural area. Clin. Infect. Dis..

[18] Berinson B., Both A., Berneking L., Christner M., Lutgehetmann M., Aepfelbacher M., Rohde H. (2021). Usefulness of BioFire FilmArray BCID2 for Blood Culture Processing in Clinical Practice. J. Clin. Microbiol..

[19] Camelena F., Pean de Ponfilly G., Pailhories H., Bonzon L., Alanio A., Poncin T., Lafaurie M., Depret F., Cambau E., Godreuil S. (2022). Multicenter Evaluation of the FilmArray Blood Culture Identification 2 Panel for Pathogen Detection in Bloodstream Infections. Microbiol. Spectr..

[20] Butler-Wu S., Davis R. Genotypic False Detections from Blood Culture Bottles: Are We Only Seeing the Tip of the Iceberg?. https://asm.org/ASM/media/Policy-and-Advocacy/BlCx-contaminating-DNA-FINAL.pdf.

[21] Saito K., Endo S., Katsumi M., Ishizawa C., Fujikawa Y., Inomata S., Toyokawa M., Kaku M. (2018). Evaluation of the FilmArray Blood Culture Identification Panel on Detection of Pathogenic Microorganisms in Positive Blood Cultures: The First Clinical Report in Japan. Jpn. J. Infect. Dis..

[22] Payne M., Champagne S., Lowe C., Leung V., Hinch M., Romney M.G. (2018). Evaluation of the FilmArray Blood Culture Identification Panel compared to direct MALDI-TOF MS identification for rapid identification of pathogens. J. Med. Microbiol..

[23] Kang C.M., Chen X.J., Chih C.C., Hsu C.C., Chen P.H., Lee T.F., Teng L.J., Hsueh P.R. (2020). Rapid identification of bloodstream bacterial and fungal pathogens and their antibiotic resistance determinants from positively flagged blood cultures using the BioFire FilmArray blood culture identification panel. J. Microbiol. Immunol. Infect..

[24] Altun O., Almuhayawi M., Ullberg M., Ozenci V. (2013). Clinical evaluation of the FilmArray blood culture identification panel in identification of bacteria and yeasts from positive blood culture bottles. J. Clin. Microbiol..

[25] Verroken A., Despas N., Rodriguez-Villalobos H., Laterre P.F. (2019). The impact of a rapid molecular identification test on positive blood cultures from critically ill with bacteremia: A pre-post intervention study. PLoS ONE.

[26] MacVane S.H., Nolte F.S. (2016). Benefits of Adding a Rapid PCR-Based Blood Culture Identification Panel to an Established Antimicrobial Stewardship Program. J. Clin. Microbiol..

